# Love actually: Is relationship status associated with dark triad personality traits and attitudes towards love?

**DOI:** 10.1016/j.heliyon.2024.e40215

**Published:** 2024-11-08

**Authors:** Agata Benfante, Marialaura Di Tella, Sara Veggi, Franco Freilone, Lorys Castelli, Georgia Zara

**Affiliations:** a Department of Psychology, University of Turin, Italy; b Department of Law, University of Turin, Italy

**Keywords:** Romantic relationship, Love attitude, Dark triad, Personality, Machiavellianism, Narcissism, Psychopathy

## Abstract

Romantic love plays a central role in the lives of individuals and influences decisions about lasting relationships such as marriage or cohabitation. To understand the dynamics of intimate relationships, both personality traits and attitudes toward love styles need to be explored. This cross-sectional study aimed to examine the possible differences between married/cohabiting and single individuals in terms of Dark Triad personality traits and attitudes towards love styles, and to investigate which of these factors can significantly predict participants’ relationship status. As a secondary objective, we analysed the presence of gender differences in the examined constructs.

A total of 1101 participants (mean age ± SD: 40.75 ± 16.07; women: 710, 64.5 %) completed the Dark Triad Dirty Dozen and the Love Attitudes Scale - Short Form. Data for this study were collected via a web-based survey.

Study's results revealed that married/cohabiting participants scored lower on all Dark Triad Dirty Dozen subscales, and they were characterised by more Eros and Agape and less Ludus, Mania, Pragma, and Storge styles compared to their single counterparts. Age, narcissism, Eros, Agape, Mania, and Storge were significant predictors of marriage/cohabitation. The final model explained 53 % of the variance, with 81 % of participants correctly categorised as married/cohabiting vs. single. Finally, men were more ludic and agapic in their love styles than women.

These findings suggest that dark personality traits and attitudes toward love styles characterise married/cohabiting and single individuals differently. Understanding these distinctions sheds light on the complexities of romantic relationships across different relationships.

## 1 Introduction

Romantic love is widely recognised as a fundamental element in people's lives, serving as a paramount motivation for committing to enduring relationships, including marriage or cohabitation [1,2]. Establishing and nurturing intimate relationships stand among the foremost challenges individuals encounter in their lives [3]. While understanding the interpersonal dynamics behind the choice of partner and the development and maintenance of these relationships is crucial, personality traits and other psychological attitudes are equally pivotal contributors to this process [1,2,[4], [5], [6]].

Personality refers to “the enduring configuration of characteristics and behaviour that comprises an individual's unique adjustment to life [ …]” and it “helps determine behaviour” [7]. Various theories have been proposed to explain personality development and structure, including attempts to distinguish between “normal”, and “abnormal”, “clinical” and “subclinical” personality and traits [8,9].

Thus, among the subclinical personalities, certain socially aversive traits have received considerable research interest. Studied separately for years, they have converged into what is known as the “Dark Triad” [10,11]. The Dark Triad personalities (i.e., Machiavellianism, narcissism and psychopathy) have been associated with a spectrum of opportunistic mating strategies, with a preference for brief, primarily sex-centred relationships, and a tendency to not engage in long-lasting relationships [5,6,[10], [12],^13^,^14^,^15^]. However, these traits were also found to be associated with “mate retention” strategies, such as a series of more or less positive behaviours aimed at retaining the partners and ensuring their fidelity in stable romantic relationships [6,16,17].

Particularly, Machiavellianism is characterised by emotional detachment, cynicism, manipulation, and good social skills aimed to obtain personal gain. This can make Machiavellian individuals less inclined to seek qualities such as trustworthiness, intimacy, and stability in intimate relationships [13,14,15,18].

People with narcissistic traits may find success in the early stages of a relationship, easily established due to their charm and overt confidence. However, characteristics of grandiosity, superiority, dominance, and reduced empathy and commitment contribute to the difficulties in forming long-term relationships. These relational difficulties are exacerbated when the narcissistic person does not receive the attention and idealisation to which they believe they are entitled [5,13,14,18,19]. Particularly, this trait is associated with so-called “sociosexuality”, as narcissistic people are more prone to a wide range of often occasional relationships [15].

Finally, manipulation and ruthlessness towards others, impulsiveness and emotional detachment, while being able to resort to glibness and superficial charm for their own benefit, are typical characteristics of individuals with psychopathic traits who are unlikely to build relationships based on trust and a genuine emotional bond [4,5,13,14,18]. Intimate and friendship relationships are therefore aimed at satisfying both sexual and, in the case of high-functioning psychopathy, affective needs [7,15].

Similar to personality traits, attitudes towards love may affect intimate relationships [2,18,20]. Particularly, the following six attitudes towards love have been identified: ‘Eros’ refers to passionate, intense and emotional love and physical attraction; ‘Ludus’ is the typical attitude of those who experience love as a game, with low commitment and scarce emotional investment; ‘Storge’ is amicable love based on sharing similar experiences and interests, rather than on passion; ‘Mania’ implies possessive and obsessive behaviour towards the partner; ‘Pragma’ is characterised by a pragmatic and rational approach to love and to the choice of a partner, who must have “convenient” characteristics; ‘Agape’ represents an approach to love that is altruistic, unconditional and selfless, with a disposition to sacrifice for the partner [2,20].

Some previous studies have shown an association between the Dark Triad traits or certain specific aspects (e.g., in the study by Blanchard and Fino [5]) and a reduced capacity to love and more negative love attitudes, indicating greater relationship difficulties [5].

In particular, previous studies seem to agree that the Ludus love style most closely describes the love attitudes of individuals with Dark Triad personalities [13]. Considering the Life History Theory, Jonason and Kavanagh [13] suggest that the Pragma love style may also reflect some of the characteristics of the Dark Triad in romantic relationships, such as the pursuit of short-term gain and advantage, or selfishness. According to this theory, individuals with Dark Triad traits are driven to allocate their energies to obtain the greatest possible benefit from relationships, including in terms of mating and reproduction [13,21]. However, with regard to the association between Dark Triad traits and other love attitude styles, the results remain inconclusive [13,18,19,22].

Studies have also suggested that there are gender differences in these two psychological characteristics [22,23]. It is known that men exhibit more Machiavellian, narcissistic and psychopathic traits than women, as evidenced in a meta-analysis [24]. Studies on attitudes towards love have shown that men generally report a more ludic style than women, while the results for other love styles are controversial [23,[25], [26], [27]]. Furthermore, these two psychological characteristics seem to be related to different relationship choices and abilities to remain in stable relationships, based on gender [23,28]. However, in some cases the results are still not consistent.

In light of previous literature, it is necessary to further investigate the possible association between the Dark Triad traits and love attitude styles, and their influence on individuals’ intimate relationships. To the best of our knowledge, no previous study has examined these variables as predictors of relationship status together or in the general Italian population.

The primary aim of this study was to examine the possible differences between married/cohabiting and single individuals in terms of Dark Triad personality traits and attitudes towards love styles, and to investigate which of these factors can significantly predict participants’ relationship status. As a secondary aim, we analysed the presence of gender differences in the constructs examined. The following hypothesised were tested.

(H1) Participants with different relationship status (married/cohabiting vs. single) would show differences in dark personality traits and preferences for love styles.
(H2) Participants' relationship status would be predicted by some Dark Triad personality traits and attitudes towards love styles.
(H3) Female and male participants would show differences in Dark Triad personality traits and attitudes toward love styles.

## 2 Methods

### 2.1 Participants

The sample consisted of 1101 participants (mean age ± SD: 40.75 ± 16.07; women: *n* = 710, 64.5 %; Italian nationality: *n* = 1089, 98.9 %) who met the following inclusion criteria: being aged 18 or older, having a minimum educational level equivalent to primary school (at least five years of education), possessing adequate knowledge of spoken and written Italian, and reporting the absence of a diagnosis of severe cognitive or psychopathological disorders, as evaluated through a yes/no question. Consequently, the exclusion criteria included: (1) being under 18; (2) having an education level lower than primary school; (3) insufficient proficiency in Italian; (4) the self-report of a severe psychiatric disorder.

### 2.2 Procedure

This cross-sectional study has been reported in accordance with the ‘Strengthening the Reporting of Observational Studies in Epidemiology’ (STROBE) guidelines, and the checklist is available as Supplementary Material [29].

Data were collected via a web-based survey from July 1, 2023, to October 31, 2023. A snowballing strategy was used in which participants were first recruited through online advertisements and encouraged to share the survey link with others. Completing the survey took an average of 15 min. Participation was voluntary and anonymous, and respondents received no compensation. Approval for this study was granted by the University of Turin Ethics Committee (protocol n. 0289029) in Italy, and the research was conducted in adherence to the Declaration of Helsinki. Written informed consent was obtained from all participants prior to administration.

### 2.3 Measures

In the current study, we focused on a subset of measures within a broader investigation. In the first part of the survey, participants were asked to provide socio-demographic information, including age, gender, educational level, sexual orientation, relationship status, and duration. Additionally, they completed the following measures.

### 2.4 The Dark Triad Dirty Dozen

The Dark Triad Dirty Dozen (DTDD) [30,31] is a 12-item scale used to assess three socially aversive personality traits: Machiavellianism, narcissism, and psychopathy. Each trait is measured using four items on a 5-point Likert scale ranging from 1 (strongly disagree) to 5 (strongly agree), where higher scores indicate higher levels of each trait. Examples of items include: “I tend to manipulate others to get my way” (Machiavellianism), “I tend to seek prestige or status” (narcissism), and “I tend to lack remorse” (psychopathy). In the validation study, the DTDD demonstrated good overall internal consistency (α = .83). Cronbach's α for the individual components were as follows: α = .72 for Machiavellianism, α = .63 for psychopathy, and α = .79 for narcissism [30]. In the current study, internal consistency coefficients were α = .87 for Machiavellianism, α = .71 for psychopathy, and α = .82 for narcissism.

### 2.5 The Love Attitudes Scale - short form

The Love Attitudes Scale - Short Form (LAS-SF) [32,33] is a 24-item questionnaire designed to assess individuals' attitudes and beliefs about romantic relationships across six love styles: Eros, Ludus, Storge, Pragma, Mania, and Agape. Participants were asked to rate their level of agreement with each statement on a 5-point scale ranging from 0 (strongly agree) to 4 (strongly disagree). The mean of the items' ratings was computed as the final score for each subscale, with lower scores reflecting a greater endorsement of that particular love style. Examples of items include: “My lover and I have the right physical chemistry between us” (Eros); “My lover would get upset if he/she knew of some of the things I've done with other people” (Ludus); “The best kind of love grows out of a long friendship” (Storge); “An important factor in choosing a partner is whether or not he/she will be a good parent” (Pragma); “When my lover doesn't pay attention to me, I feel sick all over” (Mania); “I would rather suffer myself than let my lover suffer” (Agape). The original version of LAS has the following internal consistency values (Cronbach's alpha) for each component: α = .82, α = .68, α = .84, α = .77, α = .69, α = .85, for Eros, Ludus, Storge, Pragma, Mania, and Agape [32]. In this study, the Cronbach's α were 0.81 for Eros, 0.33 for Ludus, 0.86 for Storge, 0.70 for Pragma, 0.67 for Mania, and 0.82 for Agape.

### 2.6 Statistical analyses

Statistical analyses were performed using the Statistical Package for Social Sciences (SPSS) version 29.0 (IBM SPSS Statistics for Windows, Armonk, NY, USA: IBM Corp.).

Independent-samples *t*-tests and Pearson chi-square test (χ^2^) were first used to examine group differences in continuous and categorical variables, respectively. The effect size was determined by calculating Cohen's *d* values.

In a second step, a hierarchical logistic regression analysis was conducted to examine whether sociodemographic variables (i.e., age, first block), dark triad traits (i.e., DTDD Machiavellianism, DTDD Narcissism, DTDD Psychopathy, second block), and love attitudes styles (i.e., LAS Eros, LAS Ludus, LAS Pragma, LAS Mania, LAS Agape, LAS Storge, third block) predict group membership (i.e., married/cohabiting participants vs. single participants).

The enter method was used to include variables from the predictor groups. Adjusted odds ratios and 95 % confidence intervals (Cis) were calculated for the predictors in both logistic regression models. The level of significance for all statistical tests was set at *p* < .05.

## 3 Results

Married/cohabiting vs. single individuals: differences in sociodemographic and psychological variables.

Table 1 shows sample descriptive data. The information on relationship duration provided by 615 participants shows that 5 individuals (0.8 %) were in relationships that lasted less than a year, 50 (8.1 %) reported relationships that lasted 1–3 years, and 103 (16.7 %) indicated relationships that lasted 4–10 years. The majority of those who had been in a relationship (*n* = 457; 74.3 %) reported that it had lasted more than 10 years.

**Table 1:** Sociodemographic and psychological data for the subgroups of married/cohabiting participants vs. single participants. Mean (SD) or percentage, *t*-test or chi-squared (*χ*^2^) test, and Cohen's *d* are listed.

	Married/Cohabiting (N = 615)	Single (N = 486)	Test (df)	*p*	Effect size
***Sociodemographic variables***					
Age	48.68 (13.58)	30.49 (12.92)	*t*(1099) = −22.553	<0.001	*d* = −1.369
Gender	(N = 610)	(N = 284)	*χ*^2^(1) = 12.856	<0.001	
*Male*	186 (30.2 %)	198 (40.7 %)			
*Female*	424 (68.9 %)	286 (58.8 %)			
Sexual orientation			*χ*^2^(1) = 18.741	<0.001	
*Heterosexual*	574 (93.3 %)	415 (85.4 %)			
*Other*	41 (6.7 %)	78 (14.6 %)			
***Psychological variables***					
DTDD Machiavellianism	2.44 (2.88)	3.37 (3.23)	*t*(1099) = 5.036	<0.001	*d* = 0.306
DTDD Narcissism	5.10 (3.47)	5.73 (3.77)	*t*(1099) = 2.845	0.002	*d* = 0.173
DTDD Psychopathy	3.29 (2.93)	4.57 (3.3)	*t*(1099) = 6.776	<0.001	*d* = 0.411
LAS Eros	1.17 (0.93)	1.57 (0.86)	*t*(1099) = 7.339	<0.001	*d* = 0.445
LAS Ludus	2.76 (0.71)	2.65 (0.82)	*t*(1099) = −2.193	0.014	*d* = −0.133
LAS Pragma	2.88 (0.87)	2.62 (0.97)	*t*(1099) = −4.675	<0.001	*d* = −0.284
LAS Mania	2.40 (0.87)	2 (0.86)	*t*(1099) = −7.538	<0.001	*d* = −0.458
LAS Agape	1.92 (1.01)	2.09 (0.93)	*t*(1099) = 2.914	0.002	*d* = 0.177
LAS Storge	2.69 (1.10)	2.44 (1.16)	*t*(1099) = −3.676	<0.001	*d* = −0.223

*Note:* DTDD = Dark Triad Dirty Dozen; LAS = Love Attitudes Scale.

Comparison analyses between married/cohabiting participants and single ones revealed that the former were older than the latter (*p <* .001, *d* = −1.37). Moreover, married/cohabiting participants reported lower scores on all dark personality traits [DTDD Machiavellianism (*p* < .001, *d* = 0.31), DTDD Narcissism (*p* = .002, *d* = 0.18), DTDD Psychopathy (*p* < .001, *d* = 0.41)], as well as on LAS Eros (*p* < .001, *d* = 0.45) and LAS Agape (*p* = .002, *d* = 0.18) compared to single participants. Conversely, married/cohabiting participants reported higher scores on LAS Ludus (*p* = .014, *d* = −0.13), LAS Pragma (*p* < .001, *d* = −0.28), LAS Mania (*p* < .001, *d* = −0.46), and LAS Storge (*p* < .001, *d* = −0.22) than single individuals (Table 1). This means that the married/cohabiting participants had an attitude towards love that was characterised more by Eros and Agape styles, whereas single individuals had an attitude towards love that was characterised more by Ludus, Pragma, Mania, and Storge styles.

Results of the hierarchical logistic regression analysis showed that age (OR = 1.11; 95 % CI = 1.09; 1.12), DDTD Narcissism (OR = 1.06; 95 % CI = 1.00; 1.11), LAS Eros (OR = 0.48; 95 % CI = 0.40; 0.58), LAS Mania (OR = 1.94; 95 % CI = 1.59; 2.38), LAS Agape (OR = 0.76; 95 % CI = 0.64; 0.91), and LAS Storge (OR = 1.45; 95 % CI = 1.25; 1.68) were statistically significant predictors of group membership. The final model explained 53 % of the variance, with 81 % of participants correctly categorised as married/cohabiting vs. single (Table 2).

**Table 2:** Logistic regression predicting the likelihood of being married/cohabiting vs. being single, based on sociodemographic and psychological variables.

Predictors	Model 1a				Model 2b				Model 3c			
	*B*	OR	95 % CI	Wald	*B*	OR	95 % CI	Wald	*B*	OR	95 % CI	Wald
Age	0.092	1.096	1.084–1.108	274.533∗∗∗	0.090	1.095	1.082–1.107	249.904∗∗∗	0.102	1.108	1.093–1.122	245.181∗∗∗
DTDD Machiavellianism					−0.001	0.999	0.939–1.063	0.001	0.032	1.033	0.963–1.108	0.810
DTDD Narcissism					0.032	1.032	0.984–1.083	1.717	0.053	1.055	1.000–1.113	3.861∗
DTDD Psychopathy					−0.050	0.951	0.901–1.004	3.343	−0.033	0.968	0.913–1.026	1.209
LAS Eros									−0.727	0.483	0.400–0.583	57.530∗∗∗
LAS Ludus									0.131	1.140	0.913–1.423	1.330
LAS Pragma									0.043	1.044	0.869–1.255	0.212
LAS Mania									0.664	1.943	1.588–2.378	41.541∗∗∗
LAS Agape									−0.270	0.763	0.640–0.911	9.014∗∗
LAS Storge									0.368	1.445	1.246–1.675	23.848∗∗∗

*Note:*^**a**^χ^2^(1) = 395.315, *p* < .001. Nagelkerke *R*^2^ = 0.404. ^**b**^χ^2^(4) = 400.252, *p* < .001. Nagelkerke *R*^2^ = 0.408. ^**c**^χ^2^(10) = 549.487, *p* < .001. Nagelkerke *R*^2^ = 0.526.

OR = odds ratio; CI = confidence interval; DTDD = Dark Triad Dirty Dozen; LAS = Love Attitudes Scale.

∗*p* < .05; ∗∗*p* < .01; ∗∗∗*p* < .001.

Thus, being older, having greater narcissistic traits and showing more Eros and Agape styles and less Mania and Storge style was associated with marriage/cohabitation.

### 3.1 Men and women: gender differences on love attitude style and dark triad traits

The comparison analyses between men and women are presented in Table 3.

**Table 3:** Comparisons between men and women on love attitude styles and Dark Triad personality traits.

	Women (N = 710)	Men (N = 384)	Test (df)	*p*	Effect size
DTDD Machiavellianism	2.37 (2.72)	3.72 (3.48)	t(640.14) = −6.573	<0.001	*d* = −0.448
DTDD Narcissism	5.08 (3.46)	5.91 (3.85)	t(1092) = −3.635	<0.001	*d* = −0.230
DTDD Psychopathy	3.29 (2.76)	4.91 (3.57)	t(636.17) = −7.729	<0.001	*d* = −0.527
LAS Eros	1.37 (0.93)	1.31 (0.90)	t(1092) = 1.138	0.255	*d* = 0.072
LAS Ludus	2.80 (0.72)	2.55 (0.81)	t(708.12) = 4.985	<0.001	*d* = 0.327
LAS Pragma	2.78 (0.92)	2.73 (0.93)	t(1092) = 0.944	0.345	*d* = 0.060
LAS Mania	2.25 (0.90)	2.16 (0.91)	t(1092) = 1.495	0.135	*d* = 0.095
LAS Agape	2.16 (0.95)	1.69 (0.97)	t(1092) = 7.736	<0.001	*d* = 0.490
LAS Storge	2.61 (1.14)	2.54 (1.13)	t(1092) = 1.011	0.312	*d* = −0.064

DTDD = Dark Triad Dirty Dozen; LAS = Love Attitudes Scale.

Results revealed that men reported higher scores than women in all the Dark Triad personality traits (all *p*-values <0.001). Conversely, women reported higher scores than men on the “Ludus” (*p* < .001, *d* = 0.33) and “Agape” (*p* < .001, *d* = 0.49) dimensions of the LAS. This means that men were more ludic and agapic in their love styles than women.

## 4 Discussion

The results of the present study confirmed the hypothesis tested. In fact, this study revealed significant differences in Dark Triad traits and attitude towards love styles between married/cohabiting and single participants, as well as between genders. Particularly, married/cohabiting individuals reported lower dark personality traits and more Eros and Agape styles compared to single ones. Conversely, they showed less of other styles of attitude toward love (i.e., Ludus, Mania, Pragma, and Storge) than single individuals.

The personality traits belonging to the Dark Triad are characterised by strong egocentrism, manipulative behaviour, and low morals. Individuals with these socially unfavourable traits have greater difficulty empathising with and considering the needs of others, which is essential for fostering loving interpersonal relationships [5,6,10,13,15,18], although they often succeed in attracting partners [34]. For instance, evidence on individuals with elevated psychopathic traits have reported a marked decline in relationship satisfaction over time, accompanied by higher rates of separation and divorce [35]. This deterioration in relationship quality can be attributed to several key characteristics commonly associated with psychopathy, including manipulative and exploitative behaviour patterns. Such individuals often engage in deceptive tactics, emotional detachment, and a lack of empathy, which progressively erode the foundational elements of a stable relationship, such as trust, intimacy, and mutual emotional investment. Over time, these behaviours not only create emotional distance but also foster conflict and resentment, ultimately destabilising the relationship. Moreover, the lack of remorse or accountability typical of psychopathic individuals can further exacerbate relational strains, making resolution of conflicts difficult and reducing the likelihood of long-term relationship success. Moreover, in previous studies, dark personality traits were found to be associated with a preference for short-term relationships and high levels of infidelity, which may lead to less investment in finding a stable partner and living as a couple [4,15,28]. These aspects could explain why the married/cohabiting study participants scored lower on the Dark Triad traits than the single ones.

With respect to the attitude towards love style, similarly to the previous studies [2,3,25], married/cohabiting participants showed more Eros and Agape than single people. This may be explained by the specific characteristics of the two love styles. The Eros style is associated with high intensity of love and dyadic satisfaction, and as it is also characterised by a passionate approach, it may favour remaining in the relationship as people feel involved also on a physical level [2,3,25]. The Agape style has also been positively associated with the quality of the couple's relationship [2]. Caring for and understanding the partner and prioritising the needs of the other are also part of the imagery of romantic love, which contributes to the search for and, in some cases, the maintenance of the couple [1,2].

Indeed, the other love styles could be considered suboptimal for intimate relationships and could contribute both to preferring to be single and to creating couple conditions that are not favourable to stability and durability [2,23]. For example, the Mania love style is characterised by obsessive love, coupled with jealousy and a desire for attention and affirmation from the partner. People who have this attitude towards love may have more difficulty finding partners who are willing to maintain a relationship, and they are more likely to stay single [2,3,25].

These aspects are also supported by the other results of the present study. Specifically, being older, greater narcissistic traits, having more Eros and Agape styles and less Mania and Storge styles have been associated with married/cohabiting status.

Although people with narcissistic traits have difficulty maintaining long-term relationships, according to the Agentic Model, they use intimate relationships as a self-regulation strategy. In this way, narcissists can satisfy their need for power, dominance or status enhancement by choosing partners with certain characteristics [19,36]. Differently from a previous study [3,25], in the study different love attitude styles predicted the relationship status. While it is clearer why having optimal styles, such as Eros and Agape, and less Mania predicted being in a stable relationship, it is more complex to explain why having less Storge is also associated with it. Usually, this love style is associated with a great friendship with the romantic partner, high couple satisfaction and allows for greater agreement in conflict resolution. However, by looking at the associations between the different facets of narcissism and love styles, an earlier study had shown that the most grandiose narcissism was not associated with this kind of love style [19], which is more aligned with relationships that are not functional for the narcissist's attention needs. This aspect may explain the results of the present study.

Finally, in line with results of a previous meta-analysis [24], male participants in this study reported more Dark Triad traits than women. This gender difference is probably due to both biological and socio-cultural factors that tend to encourage men to develop more malevolent, manipulative and egocentric personality traits. This could also partly explain the greater Ludus style reported by men than women in this study. Previous studies have shown that men are more likely to have a playful style of love and a lack of commitment in a relationship [23,26,27,37,38].

Regarding other gender differences, in the present study men were more agapic than women, similar to the study of Neto [39]. As a possible explanation, the Author suggested that men may be more likely than women to report being willing to sacrifice their own needs for their loved ones as a result of internalising social norms that portray them as “protectors” [39].

The present study has some limitations that must be taken into account when considering the results. First, this study relied solely on self-report instruments administered through an online survey. Second, the Ludus subscale of the LAS demonstrated low internal consistency in this study; hence, caution is advised when interpreting the results. Third, the sample was predominantly composed of women and heterosexual participants. Finally, no other psychological variables related to the constructs of interest (e.g., attachment styles) were examined. Future research should take these limitations into account by attempting to recruit a more heterogeneous population and by considering other variables or measures in combination.

Despite these limitations, the findings of the present study suggest that it is essential for psychologists and psychotherapists to carefully assess the presence of certain personality traits and love styles in their patients, in order to consider the impact of these factors on the relationship domain. This is particularly true when working with problematic couples or individuals who are struggling to establish or maintain a relationship, some of whom may not be aware that they themselves are contributing to these difficulties with their own psychological characteristics [38,40]. It may be useful to adopt an approach that helps individuals become more aware of the love styles that characterise them, while working on aspects of their relational behaviour that could mitigate the effects of socially aversive traits.

In conclusion, these results show that specific dark personality traits and attitudes towards love styles seem to characterise married/cohabiting and single people. The establishment and maintenance of intimate romantic relationships is conditioned by the psychological characteristics of individuals, shaping how they relate to and perceive others. This underscores the need for a deeper focus on these traits in both research and clinical practice to improve relational outcomes.

## CRediT authorship contribution statement

**Agata Benfante:** Writing – original draft, Investigation, Formal analysis, Conceptualization. **Marialaura Di Tella:** Writing – original draft, Methodology, Formal analysis. **Sara Veggi:** Writing – original draft, Investigation, Data curation, Conceptualization. **Franco Freilone:** Writing – review & editing, Supervision. **Lorys Castelli:** Writing – review & editing, Supervision. **Georgia Zara:** Writing – review & editing, Supervision, Conceptualization.

## Data availability statement

Data associated with this study have not been deposited into a publicly available repository and will be made available on request.

## Ethics and consent statement

This study was reviewed and approved by the Bioethics Committee of the University of Turin (Italy) with the approval number: 0289029, dated June 7th, 2023. All participants provided written informed consent to participate in the study and for their data to be published.

## Declaration of competing interest

The authors declare that they have no known competing financial interests or personal relationships that could have appeared to influence the work reported in this paper.
