# Supplementary material for: Love actually: Is relationship status associated with dark triad personality traits and attitudes towards love?
Source: Heliyon. 2024 Nov 8;10(22):e40215. doi: 10.1016/j.heliyon.2024.e40215 (PMC11693915; doi:10.1016/j.heliyon.2024.e40215)
Supplement: Multimedia component 1 [file mmc1.pdf]

Socio-demographic information

* The translation in English is in italics within parentheses.

1. Età in anni compiuti __________
(Age in completed years) __________

2. Nazionalità
(Nationality)

o Italiana o Non italiana
(o *Italian* o *Not italian)*

3. Ha un'adeguata conoscenza della lingua italiana?
(Do you have adequate knowledge of the Italian language?)

o Sì o No
(o *Yes* o *No*)

4. Ha mai sofferto in passato o attualmente soffre di un disturbo psichiatrico?
(Have you ever suffered in the past or do you currently suffer from a psychiatric disorder?)

o Sì o No
(o *Yes* o *No*)

5. Genere
(Gender)

o Uomo o Donna o Altro
(o *Male* o *Female* o *Other*)

6. Scolarità
(Educational level)

o Nessun titolo o Licenza elementare o Licenza di scuola media inferiore o Diploma di scuola media superiore o Laurea o Dottorato o altra specializzazione post-laurea
(o No formal education o Elementary school o Middle school o High school o Degree o Doctorate or other postgraduate specialization)

7. Orientamento sessuale
(Sexual orientation)

o Asessuale o Bisessuale o Dubbioso o insicuro o Eterossessuale o Fluido o Gay o Lesbica o Pansessuale o Queer o Altro o Preferisco non rispondere
(o *Asexual* o *Bisexual* o *Uncertain or insecure* o *Heterosexual* o *Fluid* o *Gay* o *Lesbian* o *Pansexual* o *Queer* o *Other* o *Prefer not to answer*)

8. Stato Civile
(Relationship status)

o Single o In una relazione sentimentale, ma non convivente o Coniugato/a o convivente
(o *Single* o *In an intimate relationship, but not cohabiting* o *Married or cohabiting*)

9. Da quanto tempo dura la sua relazione?
(*How long has your relationship been going on?)*

o Meno di un anno o 1-3 anni o 4-10 anni o Più di 10 anni
(o *Less than a year* o *1-3 years* o *4-10 years* o *More than 10 years*)

The Dark Triad Dirty Dozen (DTDD) (Jonason & Webster, 2010; Schimmenti et al., 2019)

* The translation in English is in italics within parentheses.

1. Tendo a manipolare gli altri per ottenere ciò che voglio.
(I tend to manipulate others to get my way)
o Sempre o Spesso o A Volte o Raramente o Mai
(o *Always* o *Often* o *Sometimes* o *Rarely* o *Never*)

2. Ho usato l'inganno o la menzogna per ottenere ciò che volevo.
(I have used deceit or lied to get my way)
o Sempre o Spesso o A Volte o Raramente o Mai
(o *Always* o *Often* o *Sometimes* o *Rarely* o *Never*)

3. Ho usato l'adulazione per ottenere ciò che volevo.
(I have use flattery to get my way)
o Sempre o Spesso o A Volte o Raramente o Mai
(o *Always* o *Often* o *Sometimes* o *Rarely* o *Never*)

4. Tendo a sfruttare gli altri per i miei scopi.
(I tend to exploit others towards my own end)
o Sempre o Spesso o A Volte o Raramente o Mai
(o *Always* o *Often* o *Sometimes* o *Rarely* o *Never*)

5. Tendo a non provare rimorso.
(I tend to lack remorse)
o Sempre o Spesso o A Volte o Raramente o Mai
(o *Always* o *Often* o *Sometimes* o *Rarely* o *Never*)

6. Tendo a non interessarmi della moralità delle mie azioni.
(I tend to not be too concerned with morality or the morality of my actions)
o Sempre o Spesso o A Volte o Raramente o Mai
(o *Always* o *Often* o *Sometimes* o *Rarely* o *Never*)

7. Tendo ad essere freddo/a o insensibile.
(I tend to be callous or insensitive)
o Sempre o Spesso o A Volte o Raramente o Mai
(o *Always* o *Often* o *Sometimes* o *Rarely* o *Never*)

8. Tendo ad essere cinico/a.
(I tend to be cynical)
o Sempre o Spesso o A Volte o Raramente o Mai
(o *Always* o *Often* o *Sometimes* o *Rarely* o *Never*)

9. Tendo a volere che gli altri mi ammirino.
(I tend to want others to admire me)
o Sempre o Spesso o A Volte o Raramente o Mai
(o *Always* o *Often* o *Sometimes* o *Rarely* o *Never*)

10. Tendo a volere che gli altri mi diano attenzione.

(I tend to want others to pay attention to me)

o Sempre o Spesso o A Volte o Raramente o Mai
(o *Always* o *Often* o *Sometimes* o *Rarely* o *Never*)

11. Tendo a cercare prestigio o potere.
(I tend to seek prestige or status)

o Sempre o Spesso o A Volte o Raramente o Mai
(o *Always* o *Often* o *Sometimes* o *Rarely* o *Never*)

12. Tendo ad aspettarmi favori speciali dagli altri.
(I tend to expect special favors from others)

o Sempre o Spesso o A Volte o Raramente o Mai
(o *Always* o *Often* o *Sometimes* o *Rarely* o *Never*)

Love Attitudes Scale - Short Form (LAS-SF) (Agus et al., 2018; Hendrick et al., 1998)

* The translation in English is in italics within parentheses.

1. Il/la mio/a partner ed io abbiamo tra di noi la giusta attrazione "chimica".
(My lover and I have the right physical "chemistry" between us)
o Fortemente in accordo o Moderatamente in accordo o Neutrale o Moderatamente in disaccordo o Fortemente in disaccordo
(o *Strongly agree* o *Moderately agree* o *Neutral* o *Moderately disagree* o *Strongly disagree*)

2. Sento che il/la mio/a partner ed io siamo fatti l'uno per l'altra.
(I feel that my lover and I were meant for each other)
o Fortemente in accordo o Moderatamente in accordo o Neutrale o Moderatamente in disaccordo o Fortemente in disaccordo
(o *Strongly agree* o *Moderately agree* o *Neutral* o *Moderately disagree* o *Strongly disagree*)

3. Io e il mio/a partner ci comprendiamo veramente.
(My lover and I really understand each other)
o Fortemente in accordo o Moderatamente in accordo o Neutrale o Moderatamente in disaccordo o Fortemente in disaccordo
(o *Strongly agree* o *Moderately agree* o *Neutral* o *Moderately disagree* o *Strongly disagree*)

4. Il/la mio/a partner corrisponde al mio standard ideale di bello/bellezza fisica.
(My lover fits my ideal standards of physical beauty/handsomeness)
o Fortemente in accordo o Moderatamente in accordo o Neutrale o Moderatamente in disaccordo o Fortemente in disaccordo
(o *Strongly agree* o *Moderately agree* o *Neutral* o *Moderately disagree* o *Strongly disagree*)

5. Credo che il/la mio/a partner non sarebbe ferito da ciò che non conosce di me.
(I believe that what my lover doesn't know about me won't hurt him/her)
o Fortemente in accordo o Moderatamente in accordo o Neutrale o Moderatamente in disaccordo o Fortemente in disaccordo
(o *Strongly agree* o *Moderately agree* o *Neutral* o *Moderately disagree* o *Strongly disagree*)

6. Talvolta ho dovuto tenere nascosto al/alla mio/a partner di avere altri partner.
(I have sometimes had to keep two of my lovers from finding out about each other)
o Fortemente in accordo o Moderatamente in accordo o Neutrale o Moderatamente in disaccordo o Fortemente in disaccordo
(o *Strongly agree* o *Moderately agree* o *Neutral* o *Moderately disagree* o *Strongly disagree*)

7. Il /la mio/a partner potrebbe contrariarsi se sapesse di alcune cose che ho fatto con altri/e partner.
(My lover would get upset if he/she knew of some of the things I've done with other people)
o Fortemente in accordo o Moderatamente in accordo o Neutrale o Moderatamente in disaccordo o Fortemente in disaccordo
(o *Strongly agree* o *Moderately agree* o *Neutral* o *Moderately disagree* o *Strongly disagree*)

8. Mi diverto a giocare il "gioco dell'amore" con il/la mio/a partner e con numerosi altri/e partner.
(I enjoy playing the "game of love" with a number of different partners)

○ Fortemente in accordo ○ Moderatamente in accordo ○ Neutrale ○ Moderatamente in disaccordo ○ Fortemente in disaccordo
(○ *Strongly agree* ○ *Moderately agree* ○ *Neutral* ○ *Moderately disagree* ○ *Strongly disagree*)

9. Il nostro amore è del tipo migliore, perché nasce da una lunga amicizia.
(The best kind of love grows out of a long friendship)
○ Fortemente in accordo ○ Moderatamente in accordo ○ Neutrale ○ Moderatamente in disaccordo ○ Fortemente in disaccordo
(○ *Strongly agree* ○ *Moderately agree* ○ *Neutral* ○ *Moderately disagree* ○ *Strongly disagree*)

10. Nel corso del tempo, gradualmente, la nostra amicizia si è trasformata in amore.
(Our friendship merged gradually into love over time)
○ Fortemente in accordo ○ Moderatamente in accordo ○ Neutrale ○ Moderatamente in disaccordo ○ Fortemente in disaccordo
(○ *Strongly agree* ○ *Moderately agree* ○ *Neutral* ○ *Moderately disagree* ○ *Strongly disagree*)

11. Il nostro amore è in realtà una profonda amicizia, non una misteriosa e nascosta emozione.
(Love is really a deep friendship, not a mysterious, mystical emotion)
○ Fortemente in accordo ○ Moderatamente in accordo ○ Neutrale ○ Moderatamente in disaccordo ○ Fortemente in disaccordo
(○ *Strongly agree* ○ *Moderately agree* ○ *Neutral* ○ *Moderately disagree* ○ *Strongly disagree*)

12. La nostra relazione d'amore è la più soddisfacente perché si è sviluppata da una bella amicizia.
(My most satisfying love relationships have developed from good friendships)
○ Fortemente in accordo ○ Moderatamente in accordo ○ Neutrale ○ Moderatamente in disaccordo ○ Fortemente in disaccordo
(○ *Strongly agree* ○ *Moderately agree* ○ *Neutral* ○ *Moderately disagree* ○ *Strongly disagree*)

13. Una considerazione importante che ho fatto per la scelta del/della mio/a partner è stato quanto lui/lei sarebbe stato/a in sintonia con la mia famiglia.
(A main consideration in choosing a lover is how he/she reflects on my family)
○ Fortemente in accordo ○ Moderatamente in accordo ○ Neutrale ○ Moderatamente in disaccordo ○ Fortemente in disaccordo
(○ *Strongly agree* ○ *Moderately agree* ○ *Neutral* ○ *Moderately disagree* ○ *Strongly disagree*)

14. Un fattore importante che ho considerato per la scelta del/della mio/a partner è stato se sarebbe stato/a un buon genitore.
(An important factor in choosing a partner is whether or not he/she will be a good parent)
○ Fortemente in accordo ○ Moderatamente in accordo ○ Neutrale ○ Moderatamente in disaccordo ○ Fortemente in disaccordo
(○ *Strongly agree* ○ *Moderately agree* ○ *Neutral* ○ *Moderately disagree* ○ *Strongly disagree*)

15. Una considerazione che ho fatto nella scelta del/della mio/a partner è stato quanto lui/lei avrebbe potuto condizionare la mia carriera.
(One consideration in choosing a partner is how he/she will reflect on my career)
○ Fortemente in accordo ○ Moderatamente in accordo ○ Neutrale ○ Moderatamente in disaccordo ○ Fortemente in disaccordo
(○ *Strongly agree* ○ *Moderately agree* ○ *Neutral* ○ *Moderately disagree* ○ *Strongly disagree*)

16. Prima di coinvolgermi molto con il/la mio/a partner, ho cercato di scoprire quanto il suo patrimonio genetico sarebbe stato compatibile con il mio nel caso avessimo avuto un figlio.

(Before getting very involved with anyone, I try to figure out how compatible his/her hereditary background is with mine in case we ever have children)
○ Fortemente in accordo ○ Moderatamente in accordo ○ Neutrale ○ Moderatamente in disaccordo ○ Fortemente in disaccordo
(○ *Strongly agree* ○ *Moderately agree* ○ *Neutral* ○ *Moderately disagree* ○ *Strongly disagree*)

17. Quando il/la mio/a partner non mi presta attenzione, mi sento terribilmente male.
(When my lover doesn't pay attention to me, I feel sick all over)
○ Fortemente in accordo ○ Moderatamente in accordo ○ Neutrale ○ Moderatamente in disaccordo ○ Fortemente in disaccordo
(○ *Strongly agree* ○ *Moderately agree* ○ *Neutral* ○ *Moderately disagree* ○ *Strongly disagree*)

18. Da quando sono innamorato/a del/della mio/a partner, ho difficoltà a concentrarmi su qualsiasi altra cosa.
(When I am in love, I have trouble concentrating on anything else)
○ Fortemente in accordo ○ Moderatamente in accordo ○ Neutrale ○ Moderatamente in disaccordo ○ Fortemente in disaccordo
(○ *Strongly agree* ○ *Moderately agree* ○ *Neutral* ○ *Moderately disagree* ○ *Strongly disagree*)

19. Non riesco a rilassarmi se ho il sospetto che il/la mio/a partner sia con qualcun altro.
(I cannot relax if I suspect that my lover is with someone else)
○ Fortemente in accordo ○ Moderatamente in accordo ○ Neutrale ○ Moderatamente in disaccordo ○ Fortemente in disaccordo
(○ *Strongly agree* ○ *Moderately agree* ○ *Neutral* ○ *Moderately disagree* ○ *Strongly disagree*)

20. Se per un po' di tempo il/la mio/a partner mi ignora, talvolta mi capita di fare cose stupide per provare a ottenere di nuovo la sua attenzione.
(If my lover ignores me for a while, I sometimes do stupid things to get his/ her attention back)
○ Fortemente in accordo ○ Moderatamente in accordo ○ Neutrale ○ Moderatamente in disaccordo ○ Fortemente in disaccordo
(○ *Strongly agree* ○ *Moderately agree* ○ *Neutral* ○ *Moderately disagree* ○ *Strongly disagree*)

21. Preferisco soffrire io piuttosto che vedere il /la mio/a partner soffrire.
(I would rather suffer myself than let my lover suffer)
○ Fortemente in accordo ○ Moderatamente in accordo ○ Neutrale ○ Moderatamente in disaccordo ○ Fortemente in disaccordo
(○ *Strongly agree* ○ *Moderately agree* ○ *Neutral* ○ *Moderately disagree* ○ *Strongly disagree*)

22. Non riesco a essere felice se non pongo la felicità del/della mio/a partner prima della mia.
(I cannot be happy unless I place my lover's happiness before my own)
○ Fortemente in accordo ○ Moderatamente in accordo ○ Neutrale ○ Moderatamente in disaccordo ○ Fortemente in disaccordo
(○ *Strongly agree* ○ *Moderately agree* ○ *Neutral* ○ *Moderately disagree* ○ *Strongly disagree*)

23. Di solito sono sempre pronta/o a sacrificare i miei desideri per permettere al mio/a partner di soddisfare i suoi.
(I am usually willing to sacrifice my own wishes to let my lover achieve his/hers)
○ Fortemente in accordo ○ Moderatamente in accordo ○ Neutrale ○ Moderatamente in disaccordo ○ Fortemente in disaccordo
(○ *Strongly agree* ○ *Moderately agree* ○ *Neutral* ○ *Moderately disagree* ○ *Strongly disagree*)

24. Sopporterei qualsiasi cosa per amore del/della mio/a partner.
(I would endure all things for the sake of my lover)
○ Fortemente in accordo ○ Moderatamente in accordo ○ Neutrale ○ Moderatamente in disaccordo ○ Fortemente in disaccordo
(○ *Strongly agree* ○ *Moderately agree* ○ *Neutral* ○ *Moderately disagree* ○ *Strongly disagree*)
